# Effects of Electro-Muscle Stimulation Exercise Combined with Mat Pilates on Pain, Anxiety, and Strength in Sedentary Females with Fibromyalgia: A Single-Blind Randomized Controlled Trial

**DOI:** 10.3390/jpm14070697

**Published:** 2024-06-28

**Authors:** İsmail Eseoğlu, Ali Kerim Yılmaz, Berna Anıl, Esra Korkmaz, Enes Akdemir, Coşkun Yılmaz, Lokman Kehribar, Nur Gökçe Aydın, Egemen Ermiş, Burak Yoldaş, Osman İmamoğlu

**Affiliations:** 1 Vocational School of Health Services, Dokuz Eylül University, İzmir 35340, Türkiye; ismail.eseoglu@deu.edu.tr; 2 Faculty of Yasar Doğu Sport Sciences, Ondokuz Mayıs University, Samsun 55270, Türkiye; akerim.yilmaz@omu.edu.tr (A.K.Y.); bernahltck@gmail.com (B.A.); esrkmz@gmail.com (E.K.); egemen.ermis@omu.edu.tr (E.E.); osmani@omu.edu.tr (O.İ.); 3 Kelkit Aydın Doğan Vocational School, Gümüşhane University, Gümüşhane 29600, Türkiye; coskun.yilmaz@gumushane.edu.tr; 4 Department of Orthopaedics and Traumatology, Faculty of Medicine, Dokuz Eylül University, İzmir 35340, Türkiye; lokmankehribar@gmail.com; 5 Department of Medical Ecology and Hydroclimatology, Samsun Training and Research Hospital, Samsun 55090, Türkiye; ngokceaydin@gmail.com; 6 Department of Orthopedics and Traumatology, Samsun Havza State Hospital, Samsun 55700, Türkiye

**Keywords:** fibromyalgia, exercise, sedentary, Pilates, rheumatology

## Abstract

Background and Objectives: Fibromyalgia syndrome (FM) is a chronic pain disorder that is ranked as one of the four most common rheumatological diseases in the world. This study aims to investigate the effects of an eight-week mat Pilates and electro-muscle stimulation (EMS) with combined mat Pilates exercises on pain, depression, anxiety, and strength in sedentary women. Methods: This study is a single-blind randomized controlled trial. A total of 30 sedentary female patients (Pilates (n = 15), EMS (n = 15)) diagnosed with FM were included in the study. The patients were subjected to Beck Depression (BDIs) and Anxiety Inventories (BAIs); a Fibromyalgia Impact Questionnaire (FIQ); five different Single-Leg Hop Tests (SLHTs); modified push-up (MPU), Handgrip Strength (HGS), Deep Squat (DSQ), V-Sit Flexor, bent-arm hang (BA), sit-up and Biering-Sørensen tests; and anthropometric tests before and after the 8-week exercise program. Results: The eight weeks of mat Pilates exercises combined with mat Pilates and EMS revealed significant results (*p* < 0.05) in anthropometric data (abdomen, lower abdomen, hips) (*p* < 0.05) except for the results of chest circumference measurements (*p* > 0.05). In addition, there were statistically significant positive results in BDIs, BAIs, FIQs, lower extremity (all SLHTs and DSQ), upper extremity (MPU, HGS, BA), and core (V-SIT, sit-up, Biering-Sørensen test) strength test findings (*p* < 0.05). Conclusions: Combining the mat Pilates exercises with EMS is an effective and reliable method to improve the pain, anxiety, depression, and strength of female patients diagnosed with FM.

## 1. Introduction

Fibromyalgia syndrome (FM) is a chronic pain disorder ranked as one of the four most common rheumatological diseases in the world and is characterized by widespread pain, decreased pain threshold, hyperalgesia, and allodynia [1]. This common rheumatological condition has clinical symptoms, such as fatigue, depression, anxiety, sleep disturbance, headaches, migraines, variable bowel habits, widespread abdominal pain, and urinary frequency [2]. FM is generally symptomatic because its treatment, etiology, and pathophysiology are not well understood, and various treatment methods ranging from antidepressant therapy to biofeedback and electroacupuncture are used [2]. Although its etiology and pathophysiology are not clearly understood, it is known that FM is more common among women [3].

In a study examining the physical fitness levels of patients diagnosed with FM, it was found that their muscle strength was impaired compared to that of the healthy controls [4]. Therefore, in addition to the pharmacological methods, it has been emphasized in various studies that various physical exercises, especially aerobic exercise and strength training, may be effective methods to alleviate FM-related pain and improve the patient’s physical function and general health [3,5,6,7]. Indeed, exercise therapy has been shown to be a safe and effective non-pharmacological approach to improve the physical function of patients diagnosed with FM and reduce pain [8]. Today, with the further expansion of these exercise methods, the effects of different exercise models on FM have become a focus for researchers [9,10,11], and even some exercise models such as Pilates have been added to traditional and complementary medicine practices.

Pilates has recently become a rapidly growing, popular rehabilitation activity [12]. Based on anecdotal evidence, the Pilates method has been shown to increase core strength, strengthen the spine, and help limbs regain their natural flexibility, combining mobilization, stretching, strengthening, and breathing exercises. It has been proven that it helps to achieve natural elasticity [13]. Pilates has been shown to have positive effects on low back and abdomen pain, core strength, spinal resilience, functionality, and the quality of life of patients diagnosed with FM [14,15]. Although positive effects were observed in the limited studies examining the effects of Pilates on FM, there is no control group in most of these studies [16,17]. 

In addition to the exercise methods, electro-muscle stimulation (EMS) has come to the forefront, with the development of technology. EMS increases people’s muscle strength and performance and ensures the simultaneous contraction of all the fibers forming the muscle by improving intramuscular coordination of large muscle groups in particular [18]. The literature includes studies on the effects of EMS on muscle strength and the changes it produces in people’s physical performance when combined with other training models [19]. 

Considering the positive effects of different types of exercises such as physical activity [4,8], strength [19], aerobic exercise [7], and therapeutic exercise [5] on clinical findings and some physical functions on FM, it was thought that Pilates and EMS applied in combination with Pilates would have positive effects on pain, quality of life, depression and physical, psychological, and physiological findings in patients with fibromyalgia. In addition, considering the contributions of Pilates to flexibility, proprioception, and the range of motion of the joints, the same movements performed with combined EMS by producing involuntary contractions may be effective on the feelings of muscle stiffness, pain, and fatigue due to FM. The studies combining training types with EMS generally aim to investigate the effects of EMS. Several studies showed that the exercises combined with EMS are more beneficial [20,21,22]. During the performance regeneration following training, increased blood flow accelerates the removal of muscle metabolites and reduces muscle pain [23].

Pilates and EMS exercises may be a good option in the treatment of patients diagnosed with FM as they focus on isometric contractions and cause less fatigue than aerobic exercises. Moreover, given the effects of Pilates on pain and functionality and the fact that EMS increases the overall exercise benefits, they may constitute an alternative in the treatment of FM. The scales used in the study are the tests commonly applied to patients diagnosed with FM in the literature. In addition, especially for strength tests, different strength tests that can be easily applied and commonly used in the literature were determined for the lower and upper extremity and core regions that EMS affects during exercise. Therefore, we aimed to investigate the effects of Pilates and EMS on pain, depression, strength, and anthropometric characteristics of female patients diagnosed with FM over a period of 8 weeks. The authors hypothesized that Pilates and EMS would cause different levels of improvement in female patients diagnosed with FM and that this improvement would be greater in the EMS group.

## 2. Materials and Methods

### 2.1. Experimental Design

**Figure 1:**
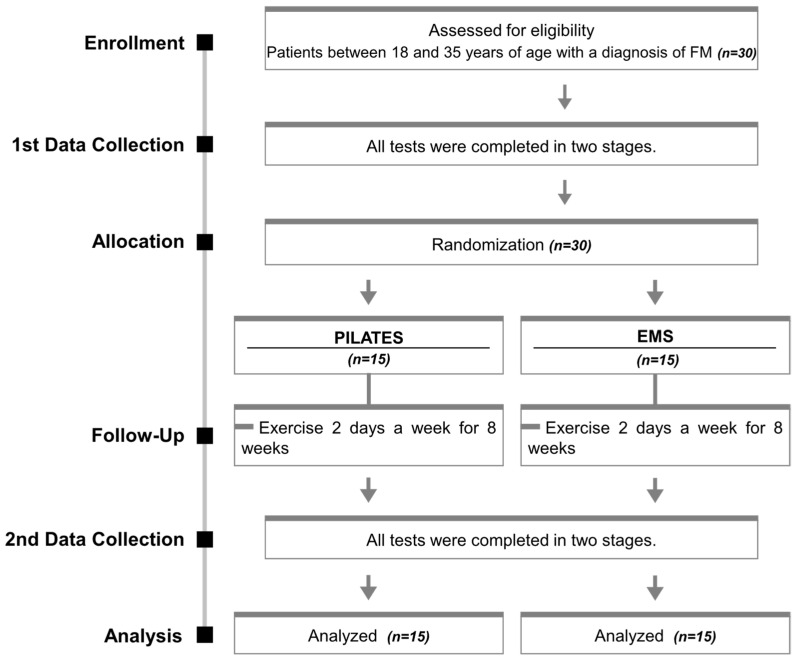
CONSORT flowchart.

This single-blind randomized controlled trial was conducted according to the recommendations of the Consolidated Standards of Reporting Trials (CONSORT) statement for randomized controlled trials by the World Health Organization (WHO) and the International Committee of Medical Journal Editors (ICMJE) (Figure 1).

### 2.2. Participants and Sample Size

A total of 30 female patients aged between 18 and 35 years who were diagnosed with fibromyalgia at the Physical Therapy and Rehabilitation Clinic of Samsun Training and Research Hospital were included in the study. An a priori test with the GPower 3.1 program was used to determine the number of participants. As a result of a power analysis sample study, it was calculated that the study could be completed with 10 patients per group (effect size: 0.80; actual power: 0.87). Participants presented to our clinic with complaints of chronic widespread pain, fatigue, sleep disturbances, tingling sensation and numbness in the hands and feet, lack of resistance to exercise, depression, feeling of waking up without rest, decreased sleep quality, and edema sensitivity.

The inclusion criteria for this study included (i) the absence of concomitant rheumatoid disease, (ii) the absence of unstable hypertension, (iii) no serious cardiopulmonary problems affecting participants’ compliance, (iv) the absence of any psychiatric disorder, (v) not being pregnant, (vi) not smoking, (vii) not following a regular exercise program, and (viii) not being diagnosed with any chronic disease accompanying fibromyalgia.

Exclusion criteria from the study included (i) concomitant rheumatoid disease, (ii) unstable hypertension, (iii) a serious cardiopulmonary problem affecting participants’ compliance, (iv) any psychiatric disorder, (v) being pregnant, (vi) smoking, (vii) following a regular exercise program, and (viii) being diagnosed with any chronic disease accompanying fibromyalgia.

### 2.3. Instruments

#### 2.3.1. Anxiety, Depression, and Pain Scales

Beck Depression and Anxiety Inventories (BDIs and BAIs, respectively): In order to investigate depression in the patients, the BDI, which consists of 21 items and yields a total score of 0–63, was applied. This inventory has a cut-off score of 17 in Turkish society. For anxiety, the BAI, which consists of 21 items and investigates somatic and cognitive anxiety complaints, was applied. This inventory is a Likert-type scale of 0–3 and has a maximum score of 63. It has a cut-off value of 17. The validity and reliability study of the inventory developed by Beck et al. was conducted by Hisli and Ulusoy et al. in Türkiye [24,25,26].

#### 2.3.2. Anthropometric Measurements

Body weight and height measurements: The subjects were asked to step on a digital scale set to zero with bare feet, wearing light clothing. The scores were recorded in kilograms. Height measurements were taken with a classical meter stick, while the subjects were in front of a plain background and were recorded in centimeters. BMI was calculated from body weight and height measurements with the following formula: “body weight (kg)/height^2^ (m^2^)”. 

Circumference measurements: Circumference measurements were taken with a non-elastic, 7 mm wide flexible tape measure while the subject was standing still with thin clothing on. Measurements were taken from the standardized thigh, arm, thorax, abdomen, lower abdomen, and hip regions and were recorded in centimeters [27].

#### 2.3.3. Lower Extremity Strength Tests

**Figure 2:**
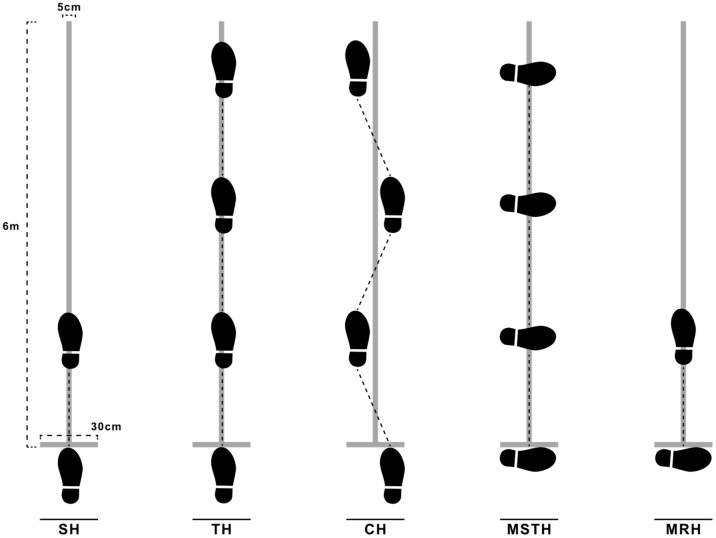
Single-Leg Hop Tests.

Single-Leg Hop Tests (SLHTs): SLHTs were applied to the single hop for distance (SH), triple hop for distance (TH), crossover triple hop for distance (CH), medial side triple hop for distance (MSTH), and medial rotation (90°) hop for distance (MRH) parameters on a single line [28]. For the line to be used in the test, a 0.3 m strip was set as the starting point, and a 6-meter-long strip with a width of 5 cm was placed vertically in the middle of it. Prior to the test, the protocol was demonstrated verbally and practically to the subjects. In each test, the subject performed one trial forward from the baseline for each test and each limb. Afterwards, they performed 3 trials for the actual test, and the scores were recorded in centimeters. An interval of 30 s was given between trials. The best score was measured from the starting line to the heel of the subject. There was no limitation for arm swinging. Following each jump, care was taken in a controlled manner to ensure that the subject was on the line for the limb tested. The tests were repeated when the subjects lost balance, when they landed on a different limb other than the one tested, or when they made an extra jump [29] (Figure 2).

Deep Squat (DSQ): In the Deep Squat test, the legs should be shoulder-width apart, and the fingertips should point straight ahead. With each command, the subjects stood in the lowest squat position they could, keeping their heels off the ground and remaining balanced. Although the subjects were entitled to three trials, they were not required to perform the remaining trials if they performed the first one successfully [30].

#### 2.3.4. Upper Extremity Strength Tests

Modified push-up test (MPU): For this test, immediately after the warm-up, the subjects, starting with their knees and elbows flexed on a mat, went into the MPU position by pushing their body backwards, extending the elbows without compromising the flexion of the knees. The number of correct movements performed within 30 s was recorded [31].

Handgrip Strength Test (HGS): For this test, the subjects were in a sitting position with shoulders in adduction and neutral rotation and elbows in a flexible position, which is the standard position recommended for HGS. For HGS, 3 trials were performed with one-minute intervals in between, and the mean scores were recorded [32].

Bent-Arm Hang Test (BA): An iron bar was placed at a height that the subjects could not reach without jumping. The subjects stood under the bar, gripping it with their thumbs under their fingers using a 2.5 cm wide apparatus. They were assisted until their chins were above the bar. They were asked to keep this position as long as they could. The test ended when the eyes of the subjects fell below the bar, and the hanging times were recorded [33].

#### 2.3.5. Core Area Strength Tests

V-Sit Flexor (Trunk Flexor) Test: Testing the endurance of the flexor muscles began with the subjects placed in a sitting position, leaning on a jig with their back at a 55-degree angle from the ground. The knees and hips were flexed at 90 °C, the arms were folded across the chest, and the feet were secured under a strap. To start, the jig was pulled back 10 cm (4 inches), and the subject tried to hold it as long as possible without disturbing the body posture. The test ended when the subject’s back touched the jig or when the subject wanted to stop [34].

Sit-Up Test: The subjects lay on a gymnastics mat in a supine position, with their hands on the back of their necks and the soles of their feet fixed on the mat. Their knees were fixed at 90° and locked. An assistant helped to fix their soles, and the number of sit-ups within 30 s was recorded [35].

Biering-Sørensen (Rear Extensor) Test: After the upper body was laid out over the end of a stretcher, the pelvis, knees, and hips were fixed. The test started after the hands were fixed crosswise on the chest. The test ended when the upper body was out of the horizontal position, when the subjects were unable to maintain their position after a verbal warning, or when the subjects requested to stop [36].

### 2.4. Procedures

This study was conducted according to the Declaration of Helsinki and Ethical approval was obtained from the Ethical Committee of the Gümüşhane University (protocol code: E-61173294-100-215335). Additionally, this trial was registered in the Iranian Clinical Trials Register (IRCT20240411061470N3) in the WHO primary registry network. Participants, the experts performing the measurements, and the experts conducting the exercise studies were fully informed about the design and purpose of the study, but the data analyzer was blinded to the study design and exercise program. This researcher carried out the data analysis after the intervention was completed, with the data table containing all the necessary data without the need to identify subjects or groups. Participants approved the informed consent form before the study and no information identifying specific patients was stored with the study data. Only the authors had access to the final study dataset and these data were anonymized and recorded throughout the study. It was anticipated that the exercise studies would not cause any harm to the participants and the patients were informed about this. All the participants were instructed to discontinue nonsteroidal anti-inflammatory medications during the study period. The participants who had started antidepressive and/or sedative medication 1 month or more before the start of the study were allowed to continue their medications and were also allowed to take acetaminophen when they had severe pain. In order to achieve a more accurate pain assessment, the patients were asked not to take acetaminophen on the morning of the evaluation day. All tests were performed at the Ondokuz Mayıs University Faculty of Yasar Doğu Sport Sciences Performance Research Laboratory and patients had a total of five laboratory visits, including a familiarization session, two visits before and two visits after the 8-week exercise course, respectively. During the first visit, age, height, weight, body mass index (BMI), and other anthropometric measurements of the subjects were recorded, and they were informed about the tests. Trial tests were also performed, and a BDI, BAI, and Fibromyalgia Impact Questionnaire (FIQ) were filled out. During the other 2 visits before the exercise, the subjects were randomized according to the type of test which they drew from the application cards and subjected to lower, upper, and core area strength tests (1- SH, CH, DSQ, MPU, HGS, and V-Sit Flexor; 2- TH, MSTH, MRH, BA, sit-up, and Biering-Sørensen test). The tests performed on the same visit were sequenced to include different areas (lower, upper, and core) and sufficient rest intervals were provided between the tests. Following the first measurements, all participants were randomly allocated into “intervention A” and “intervention B” using a randomization list by a researcher with the measurement results. After allocation, groups A and B were designated as mat Pilates and EMS, respectively. The mat Pilates and EMS groups were subjected to the same exercise protocol 2 days a week for 8 weeks (Table 1). Participants’ attendance to the exercise program in both groups was recorded by the persons conducting the exercise program through a follow-up list during the entire exercise program period. The study was completed with the initial group of participants and no participant stopped the study during the process. A device was introduced to the group that would apply EMS, and they were informed about the content. During the 2 visits following the 8-week exercise course, the subjects were randomized again using the application cards, and all tests were repeated. All questionnaires were also filled out before the tests. The content and scope of the exercise were designed to affect the lower, upper, and core strengths. In order to ensure complete rest, the interval between tests was set to 48 h. All measurements and exercises were conducted at the same time of the day (14:00–16:00). The criteria for discontinuation of the research included the following: (i) a participant withdraws consent to participate in the study, (ii) experts decide that it is not appropriate for the participant to continue with the exercise, and (iii) failure to follow the study protocol.

#### Interventions

Electro-Muscle Stimulation (EMS)

**Figure 3:**
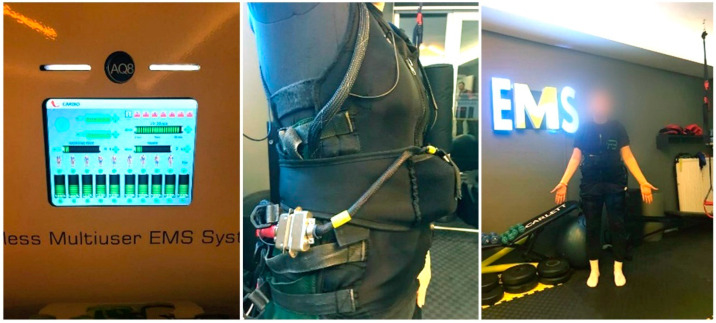
EMS device and vest.

A device (AQ8 Wireless Multiuser EMS System^®^, Lexter Microelectronic, Madrid, Spain) was introduced to the group that would apply EMS, and information about the content was given. Before the subjects were put on the specially prepared vests, water was sprayed on the main muscle groups that matched the current-conducting apparatus inside the vests (Figure 2). EMS currents were applied to the subjects for 20 min in cardio mode (low-frequency currents ranging between 1 and 1000 Hz, constantly changing direction) in the form of 9 s current/2 s rest. The muscles stimulated with EMS were as follows: gluteus maximus, quadriceps, and hamstring in the lower extremity, biceps brachii, triceps brachii, pectoralis major, and teres major in the upper extremity, and rectus abdominis and latissimus dorsi for the core (Figure 3).

### 2.5. Statistical Analyses

The SPSS 21 program was used in the statistical analysis of the study. The results are presented as mean and standard deviation. A Shapiro–Wilk test was used for normality, and a Levene test was used for homogeneity assumptions. To compare the paired groups (Pre/Post), a paired sample t-test was used, while inter-group comparisons (mat Pilates and EMS) were conducted with a repeated measures ANOVA 4 × 2 mixed model test. In addition, in the comparison of the paired groups, effect sizes were found according to Cohen’s d effect size (M2 − M1)⁄SDpooled). According to this formula, a d value of <0.2 was defined as a weak effect, a d value of 0.5 was defined as a moderate effect, and a d value of >0.8 was defined as a strong effect. The statistical results were evaluated at a significance level of *p <* 0.05.

## 3. Results

There was no significant difference between the groups in terms of the average age of the patients from whom lateral meniscal tissues were obtained (*p* = 0.772) (Table 2). When the BMI values were examined, although the patients in Group 2 were heavier, there was no statistically significant difference between the groups (*p* = 0.079) (Table 2).

There were pre- and post-statistically significant differences in the EMS group and Pilates group with respect to the BAI, BDI, and FIQ scores (*p* < 0.05). The inter-group comparison revealed that there were significant differences between EMS and Pilates groups with respect to the BAI, BDI, and FIQ scores (*p <* 0.05) (Table 3).

In the pre- and post-anthropometric measurements of the groups, statistically significant differences were found in the right (*p* = 0.022) and left (*p* = 0.022) arm circumferences in the Pilates group, and in the abdomen, lower abdomen, and hip parameters in the EMS and Pilates groups (*p* < 0.05). In inter-group comparisons, differences were found between EMS and Pilates groups in terms of abdomen, lower abdomen, and hip parameters (*p* < 0.05) (Table 4).

The pre- and post-test effect sizes for the thigh parameter were 0.11 (R) and 0.05 (L) in the EMS group and 0.04 (R) and 0.04 (L) in the Pilates group, respectively. For the arm parameter, these values were 0.78 (R) and 0.62 (L) in the EMS group and 0.49 (R) and 0.46 (L) in the Pilates group, respectively.

Table 5 presents the pre- and post-comparisons of the Single-Leg Hop Tests. Significance was found for all parameters in EMS and Pilates groups (*p* < 0.05). Significant differences were found between EMS and Pilates groups for TH, CH, MSTH, and MRH parameters in right-side comparisons between groups (*p* < 0.05). There were significant differences between EMS and Pilates groups for SH, TH, CH, and MSTH tests (*p* < 0.05).

The pre- and post-test effect sizes in the SH parameter were 1.22 (R) and 1.44 (L) in the EMS group and 1.42 (R) and 1.11 (L) in the Pilates group, respectively. In the TH parameter, these values were 0.93 (R) and 0.70 (L) in the EMS group and 0.79 (R) and 0.85 (L) in the Pilates group, respectively. In the CH parameter, these values were 1.07 (R) and 1.34 (L) in the EMS group and 0.95 (R) and 0.92 (L) in the Pilates group, respectively. In the MSTH parameter, these values were 1.40 (R) and 0.93 (L) in the EMS group and 0.51 (R) and 0.42 (L) in the Pilates group, respectively. In the MRH parameter, these values were 1.37 (R) and 0.72 (L) in the EMS group and 1.19 (R) and 1.97 (L) in the Pilates group (Table 5), respectively.

The pre- and post-evaluation of the lower and upper extremity strength tests for the EMS and Pilates groups reveal that there were statistically significant differences in all the parameters in the between groups (*p <* 0.05). As for the inter-group comparison, there were statistically significant differences between the EMS and Pilates groups only for the Sit-up parameter (*p <* 0.05) (Table 6).

The pre- and post-test effect sizes in the Handgrip parameter were 1.03 (R) and 1.49 (L) in the EMS group and 1.04 (R) and 1.20 (L) in the Pilates group (Table 6), respectively.

## 4. Discussion

This study examined the effects of mat Pilates and EMS exercises on the depression, anxiety, anthropometric, and strength characteristics of female patients diagnosed with FM, revealing major findings at different levels. These findings were that both Pilates and EMS exercises significantly reduced the pain, depression, and anxiety scores; that there were positive decreases in anthropometric data; and that they significantly improved their lower and upper extremity and core strength. 

Other researchers have investigated the effects of different types of exercises on many factors, such as pain, depression, anxiety, and the quality of life of patients diagnosed with FM. Although there are enough studies in the literature examining the effects of different forms of Pilates on patients diagnosed with FM [17,37], there are no studies evaluating the results of EMS exercises. Previous studies have shown that physical exercise is of great importance in the treatment of FM. Sieczkowska et al. [38], in a study on mostly female fibromyalgia patients, found that the overall quality of life of patients who exercised was significantly better than those who did not exercise and emphasized the importance of exercise in reducing fibromyalgia symptoms. Macfarlane et al. [39], in their recommendation revision of EULAR FM management, strongly recommended exercise particularly due to its ease for people with pain to improve their quality of life, physical functions, and well-being, and also because of its low cost and lack of safety problems. Medeiros et al. [16] revealed the positive effects of mat Pilates and aquatic exercise on the pain, function, sleep, quality of life, and fear avoidance of women diagnosed with FM. Although other studies examining the effects of different Pilates forms on FM have different limitations, they all concluded that Pilates has positive effects, particularly on the pain and related factors of patients diagnosed with FM [17,37,40]. Other studies examining the effects of different forms of exercise (aquatic, resistance training, dance, and aerobic exercise) on patients diagnosed with FM revealed positive results, as those on Pilates did [41,42,43,44]. However, the commonality of these studies was that they tried to find out which exercise form was the most effective method for patients diagnosed with FM. Accordingly, this study revealed similar mat Pilates and EMS exercise results in parameters such as pain and anxiety. In view of the positive effects of Pilates form on FM, as Lima et al. [45] argued, there is a balance between inhibition and excitation in the central nervous system, and this balance determines whether exercise increases analgesia or pain. Therefore, the most logical interpretation is that various factors, such as physical condition, physical activity, and injury or pain, affect this balance. There are several studies supporting this interpretation [16,45]. In this study, there was an improvement, particularly in the FIQ scores of the EMS group in regard to the effect size (ES) = 2.16, which was ES = 1.43 in the Pilates group. Better BAI, BDI, and FIQ scores following exercises performed with EMS compared to mat Pilates can be attributed to the following: the muscles provide intramuscular coordination during EMS exercises and reduce the inhibition resulting from FM by simultaneously contracting all the fibers that make up the muscles through electrical stimulation, which makes the muscles more active and increases stimulation and circulation. In fact, other researchers have suggested that increased blood flow during EMS exercise may lead to the removal of muscle metabolites and the reduction in muscle pain [23]. 

In studies evaluated with different approaches, the level of metabolic, physiological, and morphological changes in EMS, in which involuntary contractions are provided as opposed to voluntary contractions, were evaluated and these results supported our current research results. In a study, the effects of EMS applied to large lower extremity muscles on cerebral blood flow and the changes in these effects on the internal carotid and vertebral arteries were observed. The research results emphasized that EMS accelerated cerebral blood flow, but did not increase blood flow in the vertebral arteries [46]. Another study conducted in rats examined the relationship between metabolic parameters and plasma meteorin (Metrnl) concentrations in white and brown adipose tissues of chronic endurance exercises combined with EMS and found that the changes, especially in brown adipose tissue, were associated with mitochondrial biogenesis. The research results suggested that EMS may be an effective method in preventing metabolic diseases by altering white and brown adipose tissues and increasing Mertnl concentrations [47].

It is clearly emphasized in the literature that different exercise forms have positive effects on strength and power-based performance parameters, particularly in sedentary women. Other studies have reported the positive effects of EMS exercises combined with normal exercise protocols on strength and similar performance components [20,21]. This study revealed significant increases in all the lower, upper, and core strength tests in the mat Pilates and EMS exercise groups. However, this increase was not distinguishable when these two exercise groups were compared. Other similarly designed randomized controlled studies showed that exercise groups diagnosed with FM have much greater strength development compared to those who do not perform any exercise [48,49]. However, a comparison of individuals diagnosed with FM who were exposed to strength exercises and healthy individuals revealed similar strength improvements [50,51,52]. In this study, SLHTs were used to measure lower extremity functional strength. Although a similar test protocol is not found in the literature for patients diagnosed with FM, there are studies measuring lower extremity strength. Valkeinen et al. [51] observed a higher increase in strength in the group diagnosed with FM compared to that of the control group following strength training; they studied women with and without a diagnosis of FM, but this increase revealed similar rates in muscle activation. Hakkinen et al. [49] also reported similar results. Another study compared the effects of strength training in women diagnosed with FM and healthy controls and observed a significant increase in muscle strength, which is similar to our study [53]. In a similar study, Andrade et al. [54] found that 8-week strength training provided significant improvement in strength-related functional capacity in fibromyalgia patients. Almost all of the studies examining the effects of exercise programs on strength in patients diagnosed with FM have produced significant results. In fact, the effects of mat Pilates and EMS exercises on strength are similar in this study. These results led to the conclusion that this patient group consisted of sedentary women and that both exercise models showed similar development in an 8-week period. However, exercise programs planned for longer terms may produce different results. In addition, the improvement in muscle strength as a result of exercises reduced the inhibition of the muscles resulting from FM, making the muscles more active, and the level of pain decreased with the improvement in strength. 

In this study, significant improvements were observed in the anthropometric data of the patients in both the mat Pilates and EMS groups. As reported in other studies, both regular strength and aerobic exercises of different durations have positive effects on the anthropometric data [55,56]. According to these results, we believe that the increase in muscle ratio and decrease in fat ratio as a result of exercise in sedentary women also affected the anthropometric, strength and pain, depression, and anxiety results.

This research had several important limitations. The most is the lack of healthy exercise groups in the study. Furthermore, although it is known that FM is more common in women, the fact that only female participants were included in the study and male patients were not included is an important limitation. Another important limitation is that we analyzed a relatively small group of patients in a limited age range (18–35). In addition, there was no intervention in the diets of patients. Although the most appropriate tests and scales for patients diagnosed with FM were used after the literature search, parameters, such as a more detailed patient history (training history, occupation, family history of FM or any rheumatological disease, VAS pain score, etc.), were not included in this study. In addition, another limitation is that the variable follow-up periods of the patients were not taken into account in the analyses, although attention was paid to the fact that the patients were diagnosed at least 6 months before. Another limitation is the possibility of placebo effects on the results since measurements were taken immediately after the exercise program in both groups. Finally, although an 8-week exercise program was conducted in the present study, there were no interim measurements.

In addition, in future studies, it is important to follow up for 12–18 months between the exercise period and the data collection period, taking into account possible placebo effects in the measurement results. Additionally, it is important to investigate biomarkers that show the results more clearly and objectively in order to reduce the risks of unscientific clinical results for the validity of the results. Since this study is the first to examine the results of mat Pilates combined with EMS for female patients diagnosed with FM, future studies with longer periods and different exercise models will provide clearer results and present a more explicit picture of the therapeutic results of EMS exercises.

## 5. Conclusions

In light of all the above-mentioned information, the results of this study revealed that mat Pilates exercises combined with EMS provide an effective and reliable method of improving the pain, anxiety, depression, and strength of female patients diagnosed with FM. However, the experience of the trainers and their close follow-ups should also be considered in this respect. 

## Figures and Tables

**Table 1:** Unit training programs of mat Pilates and EMS groups.

Exercise	Rest
Jump rope—30 s × 2	15 s intervals between all exercises, 30 s rest between sets.
Overhead stand crunch—30 s × 2	
Overhead side bends—30 s × 2	
Squat—30 s × 2	
Sumo squat—30 s × 2	
Overhead lunge—30 s × 2	
Side lunge—30 s × 2	
Sit-up—30 s × 2	
Reverse sit-up—30 s × 2	
Single-leg drop—30 s × 2	
Flutter kicks—30 s × 2	
V-sit—30 s × 2	
Leg hold—30 s × 2	
Reverse leg lift—30 s × 2	
Heel touch—30 s × 2	
Point single-leg bridge drop—20 s × 2	
Plank—30 s × 2	

**Table 2:** Descriptive information for all groups.

	EMS				PILATES				*t*	*p* Value
	Mean	SD	Min.	Max.	Mean	SD	Min.	Max.		
Age (year)	27.50	5.17	21	35	25.80	5.09	21	35	1.563	0.228
Height (cm)	163.50	5.30	155	170	163.30	5.17	156	170	0.0670	0.520
Weight (kg)	62.66	10.61	53	87	62.48	13.68	51	90	0.494	0.616
BMI (kg/m^2^)	23.42	3.62	18.34	31.20	22.52	4.32	18.42	31.14	0.740	0.487

SD: standard deviation; Min: minimum; Max: maximum; BMI: body mass index.

**Table 3:** Beck Anxiety Inventory, Beck Depression Inventory, and Fibromyalgia Impact Questionnaire scores for the groups, pre- and post-comparison.

	EMS				PILATES				ANOVA Results *p* Value
	Pre	Post			Pre	Post			
	Mean ± SD	Mean ± SD	*ES*	*p* Value	Mean ± SD	Mean ± SD	*ES*	*p* Value	
BAI	23.55 ± 4.30	15.41 ± 2.69	2.27	**<0.001 ^1^**	21.60 ± 4.48	15.94 ± 4.89	1.21	**0.009 ^2^**	**<0.005 ^3^**
BDI	16.30 ± 4.87	9.29 ± 2.58	1.80	**<0.001 ^1^**	16.22 ± 5.08	8.53 ± 3.36	1.79	**<0.001 ^2^**	**<0.001 ^3^**
FIQ	50.86 ± 9.35	34.04 ± 5.80	2.16	**<0.001 ^1^**	52.95 ± 10.39	39.07 ± 9.01	1.43	**<0.001 ^2^**	**<0.001 ^3^**

Note: Bold values indicate statistical significance (*p* < 0.05). ^1^ Paired sample t-test for EMS group pre- and post-measurements. ^2^ Paired sample t-test for Pilates group pre- and post-measurements. ^3^ Repeated measures ANOVA. SD: standard deviation; ES: effect size; BAI: Beck Anxiety Inventory; BDI: Beck Depression Inventory; FIQ: Fibromyalgia Impact Questionnaire.

**Table 4:** Pre- and post-comparison of anthropometric data of groups.

	EMS				PILATES			
	R		L		R		L	
	Pre	Post	Pre	Post	Pre	Post	Pre	Post
(cm)	Mean ± SD	Mean ± SD	Mean ± SD	Mean ± SD	Mean ± SD	Mean ± SD	Mean ± SD	Mean ± SD
Thigh	57.00 ± 4.32	57.40 ± 2.99	57.00 ± 4.74	56.80 ± 3.05	56.10 ± 7.45	56.40 ± 6.82	55.50 ± 6.69	56.70 ± 7.47
Arm	27.70 ± 2.31	30.10 ± 3.70	28.00 ± 2.16	29.40 ± 2.37	26.70 ± 2.67	**28.20 ± 3.39 ^2^**	27.20 ± 2.44	**28.50 ± 3.17 ^2^**
	**EMS**				**PILATES**			
	**Pre**	**Post**	**ES**	***p* Value**	**Pre**	**Post**	**ES**	***p* Value**
	**Mean ± SD**	**Mean ± SD**			**Mean ± SD**	**Mean ± SD**		
Thorax	88.10 ± 7.26	89.40 ± 6.13	0.19	0.565	85.60 ± 12.46	91.00 ± 11.25	0.45	0.203
Abdomen	81.40 ± 8.82	75.10 ± 8.33	0.73	**<0.001 ^1^**	80.80 ± 10.65	75.20 ± 11.03	0.52	**<0.001 ^2^**
Lower Abdomen	94.20 ± 5.96	87.60 ± 5.44	1.16	**<0.001 ^1^**	94.50 ± 12.37	88.90 ± 11.59	0.47	**<0.001 ^2^**
Hip	102.60 ± 5.62	93.40 ± 4.72	1.77	**<0.001 ^1^**	100.90 ± 9.79	92.80 ± 10.72	0.79	**<0.001 ^2^**

Note: Bold values indicate statistical significance (*p* < 0.05). ^1^ Paired sample *t*-test for EMS group pre- and post-measurements. ^2^ Paired sample *t*-test for Pilates group pre- and post-measurements. R: right; L: left; SD: standard deviation; ES: effect size.

**Table 5:** Comparison of pre- and post-Single-Leg Hop Test results for groups.

	EMS				PILATES			
	R		L		R		L	
	Pre	Post	Pre	Post	Pre	Post	Pre	Post
(cm)	Mean ± SD	Mean ± SD	Mean ± SD	Mean ± SD	Mean ± SD	Mean ± SD	Mean ± SD	Mean ± SD
SH	101.80 ± 18.83	**129.80 ± 26.36 ^1^**	96.40 ± 17.14	**125.80 ± 23.15 ^1^**	100.60 ± 13.84	**124.30 ± 19.11 ^2^**	94.80 ± 18.09	**118.50 ± 24.30 ^2^**
TH	346.90 ± 59.91	**402.20 ± 58.59 ^1^**	321.70 ± 64.63	**363.30 ± 53.88 ^1^**	320.30 ± 44.40	**362.30 ± 60.41 ^2^**	291.00 ± 68.80	**349.30 ± 68.78 ^2^**
CH	296.80 ± 50.41	**347.80 ± 44.30 ^1^**	296.50 ± 41.00	**348.90 ± 37.25 ^1^**	278.70 ± 48.96	**336.70 ± 71.27 ^2^**	287.70 ± 53.43	**339.70 ± 58.98 ^2^**
MSTH	245.70 ± 36.35	**295.20 ± 34.22 ^1^**	249.70 ± 38.44	**291.10 ± 49.97 ^1^**	257.90 ± 57.91	**287.20 ± 56.10 ^2^**	250.60 ± 62.33	**279.70 ± 74.53 ^2^**
MRH	108.40 ± 17.83	**134.80 ± 20.74 ^1^**	104.60 ± 15.81	129.60 ± 46.19	105.00 ± 18.76	**127.10 ± 18.34 ^2^**	102.70 ± 15.91	**137.30 ± 19.08 ^2^**

Note: Bold values indicate statistical significance (*p* < 0.05). ^1^ Paired sample *t*-test for EMS group pre- and post-measurements. ^2^ Paired sample *t*-test for Pilates group pre- and post-measurements. R: right; L: left; SD: standard deviation; SH: single hop for distance; TH: triple hop for distance; CH: crossover triple hop for distance; MSTH: medial side triple hop for distance; MRH: medial rotation (90°) hop for distance.

**Table 6:** A comparison of pre- and post-evaluation of upper extremity and core strength tests of Ems and Pilates groups.

	EMS				PILATES				ANOVA Results *p* Value
	Pre	Post			Pre	Post			
	Mean ± SD	Mean ± SD	ES	*p* Value	Mean ± SD	Mean ± SD	ES	*p* Value	
MPU (rep)	5.00 ± 2.67	10.40 ± 3.98	1.59	**<0.001 ^1^**	4.30 ± 2.11	9.40 ± 3.13	1.91	**<0.001 ^2^**	**0.012 ^3^**
BA (s)	9.50 ± 3.96	13.80 ± 3.43	1.16	**<0.001 ^1^**	7.60 ± 2.17	11.90 ± 4.84	1.15	**0.005 ^2^**	**0.008 ^3^**
DSQ (rep)	14.40 ± 3.06	18.20 ± 2.82	1.29	**0.001 ^1^**	13.40 ± 2.12	18.60 ± 4.50	1.48	**<0.001 ^2^**	**<0.001 ^3^**
V-SIT (s)	50.50 ± 21.97	71.80 ± 28.78	0.83	**0.001 ^1^**	44.20 ± 17.43	69.50 ± 40.30	0.81	**0.015 ^2^**	**<0.001 ^3^**
Sit-Up (rep)	11.80 ± 2.04	16.10 ± 1.85	2.21	**<0.001 ^1^**	13.00 ± 2.11	16.10 ± 2.42	1.37	**<0.001 ^2^**	**<0.001 ^3^**
Biering-Sørensen (s)	46.30 ± 17.58	61.60 ± 15.62	0.92	**<0.001 ^1^**	41.30 ± 10.93	53.60 ± 15.98	0.90	**0.001 ^2^**	**<0.001 ^3^**
	**EMS**				**PILATES**				
	**R**		**L**		**R**		**L**		
	**Pre**	**Post**	**Pre**	**Post**	**Pre**	**Post**	**Pre**		**Post**
	**Mean ± SD**	**Mean ± SD**	**Mean ± SD**	**Mean ± SD**	**Mean ± SD**	**Mean ± SD**	**Mean ± SD**		**Mean ± SD**
HGS (kg)	24.84 ± 3.74	**29.03 ± 4.39 ^2^**	21.39 ± 2.21	**25.92 ± 3.69 ^2^**	24.69 ± 2.76	**28.11 ± 3.76 ^2^**	21.84 ± 3.07		**25.52 ± 3.07 ^2^**

Note: Bold values indicate statistical significance (*p* < 0.05). ^1^ Paired sample t-test for EMS group pre- and post-measurements. ^2^ Paired sample *t*-test for Pilates group pre- and post-measurements. ^3^ Repeated measures ANOVA. R: right; L: left; SD: standard deviation; ES: effect size; rep: repetition; s: second; MPU: modified push-up; BA: bent-arm hang; DSQ: Deep Squat; HGS: Handgrip Strength.

## Data Availability

The datasets used and/or analyzed during the current study are available from the corresponding author upon reasonable request.
